# Achieving High Accuracy in Predicting the Probability of Periprosthetic Joint Infection From Synovial Fluid in Patients Undergoing Hip or Knee Arthroplasty: The Development and Validation of a Multivariable Machine Learning Algorithm

**DOI:** 10.7759/cureus.51036

**Published:** 2023-12-24

**Authors:** Pearl R Paranjape, Van Thai-Paquette, John L Miamidian, Jim Parr, Eyal A Kazin, Alex McLaren, Krista Toler, Carl Deirmengian

**Affiliations:** 1 Department of Diagnostics Research and Development, Zimmer Biomet, Warsaw, USA; 2 Department of Data Science and Machine Learning, Zimmer Biomet, Swindon, GBR; 3 Department of Orthopaedic Surgery, University of Arizona College of Medicine - Phoenix, Phoenix, USA; 4 Department of Orthopaedic Surgery, The Rothman Orthopaedic Institute, Philadelphia, USA; 5 Department of Orthopaedic Surgery, Thomas Jefferson University, Philadelphia, USA

**Keywords:** artificial intelligence (ai), probability prediction, algorithmic analysis, multianalyte assay, machine learning algorithm, pji diagnosis, hip arthroplasty, knee arthroplasty, synovial fluid analysis, periprosthetic joint infection

## Abstract

Background and objective

The current periprosthetic joint infection (PJI) diagnostic guidelines require clinicians to interpret and integrate multiple criteria into a complex scoring system. Also, PJI classifications are often inconclusive, failing to provide a clinical diagnosis. Machine learning (ML) models could be leveraged to reduce reliance on these complex systems and thereby reduce diagnostic uncertainty. This study aimed to develop an ML algorithm using synovial fluid (SF) test results to establish a PJI probability score.

Methods

We used a large clinical laboratory's dataset of SF samples, aspirated from patients with hip or knee arthroplasty as part of a PJI evaluation. Patient age and SF biomarkers [white blood cell count, neutrophil percentage (%PMN), red blood cell count, absorbance at 280 nm wavelength, C-reactive protein (CRP), alpha-defensin (AD), neutrophil elastase, and microbial antigen (MID) tests] were used for model development. Data preprocessing, principal component analysis, and unsupervised clustering (K-means) revealed four clusters of samples that naturally aggregated based on biomarker results. Analysis of the characteristics of each of these four clusters revealed three clusters (n=13,133) with samples having biomarker results typical of a PJI-negative classification and one cluster (n=4,032) with samples having biomarker results typical of a PJI-positive classification. A decision tree model, trained and tested independently of external diagnostic rules, was then developed to match the classification determined by the unsupervised clustering. The performance of the model was assessed versus a modified 2018 International Consensus Meeting (ICM) criteria, in both the test cohort and an independent unlabeled validation set of 5,601 samples. The SHAP (SHapley Additive exPlanations) method was used to explore feature importance.

Results

The ML model showed an area under the curve of 0.993, with a sensitivity of 98.8%, specificity of 97.3%, positive predictive value (PPV) of 92.9%, and negative predictive value (NPV) of 99.8% in predicting the modified 2018 ICM diagnosis among test set samples. The model maintained its diagnostic accuracy in the validation cohort, yielding 99.1% sensitivity, 97.1% specificity, 91.9% PPV, and 99.9% NPV. The model's inconclusive rate (diagnostic probability between 20-80%) in the validation cohort was only 1.3%, lower than that observed with the modified 2018 ICM PJI classification (7.4%; p<0.001).

The SHAP analysis found that AD was the most important feature in the model, exhibiting dominance among >95% of "infected" and "not infected" diagnoses. Other important features were the sum of the MID test panel, %PMN, and SF-CRP.

Conclusions

Although defined methods and tools for diagnosis of PJI using multiple biomarker criteria are available, they are not consistently applied or widely implemented. There is a need for algorithmic interpretation of these biomarkers to enable consistent interpretation of the results to drive treatment decisions. The new model, using clinical parameters measured from a patient’s SF sample, renders a preoperative probability score for PJI which performs well compared to a modified 2018 ICM definition. Taken together with other clinical signs, this model has the potential to increase the accuracy of clinical evaluations and reduce the rate of inconclusive classification, thereby enabling more appropriate and expedited downstream treatment decisions.

## Introduction

Periprosthetic joint infection (PJI) can be difficult to diagnose following total joint arthroplasty (TJA). With a reported incidence ranging from 1 to 2% following primary procedures and 3 to 10% following revision arthroplasty, PJI is the leading cause of total knee arthroplasty revision and the third most common cause of total hip arthroplasty revision [1]. The Musculoskeletal Infection Society (MSIS) first proposed a definition for PJI in 2011 with a multiple-criterion diagnostic scoring system [2]. Since then, the concept of PJI definition and scoring systems has undergone iterative revisions through the International Consensus Meeting (ICM), European Bone and Joint Infection Society (EBJIS), Infectious Diseases Society of America (IDSA), and others [2-6]. The 2018 ICM definition assigns a points-based score to patients using multiple preoperative and intraoperative biomarker thresholds. Based on the total score, the patient is classified as "infected", "not infected", or "inconclusive" [4]. Although academic surgeons familiar with PJI research may be able to apply the 2018 ICM scoring system, its complexity may hinder widespread adoption in clinical practice [7,8]. Recent studies have found that clinician reliance on laboratory-reported normal thresholds may lead to false-positive interpretation [9], and physician use of multiple criteria to diagnose PJI was inferior to the alpha-defensin (AD) test alone [10].

The 2018 ICM scoring system improved on the sensitivity and specificity of the 2011 and 2013 definitions. However, the scoring system remains dependent on rigid binary diagnostic classification thresholds [11]. Furthermore, Sigmund, et al. reported that 26% of cases were diagnosed as “inconclusive” preoperatively by the 2018 ICM criteria [12], and previous work using a modified 2018 ICM applied to a large cohort of synovial fluid (SF) samples reported an inconclusive rate of 7.5% [13]. As the biomarker profile indicative of PJI can vary from one individual to another, a more robust schema for PJI diagnostic scoring is needed.

Classification machine learning (ML) techniques can be used to develop algorithms to predict diagnostic categories based on data features [14,15]. Typically, a dataset can be labeled with the diagnosis and then split into a training set and test set, which is utilized to develop an ML model for diagnosis. For diseases with a definitive gold standard method, this supervised learning approach can be utilized to match the ML model results with the gold standard results [16]. However, for complex datasets without definitive classification categories, supervised training is limited in its effectiveness, as it heavily relies on labeled training data and predefined output classes, making it challenging to capture nuanced patterns and relationships within intricate data structures [14]. In such cases, unsupervised learning techniques, such as dimensionality reduction by principal component analysis (PCA) and clustering (K-means), may prove more suitable for uncovering hidden structures and extracting meaningful insights from the data [15].

Furthermore, by leveraging combinatorial usage of unsupervised and supervised ML techniques to synergistically choose the most informative features and subsequently train a classification model, the overall predictive performance and robustness of the model can be enhanced, enabling a more nuanced understanding of complex datasets and improving the ability to accurately classify and interpret diverse patterns within the data [17,18]. Such hybrid and semi-supervised ML approaches have been extensively studied and proven to be highly effective in medical diagnosis [19]. For example, in a study by Zheng et al. [20], a hybrid method was applied. K-means clustering was employed to analyze the data patterns distinguishing between benign and malignant breast cancer tumors. The identified patterns were then used to train a support vector machine (SVM) classifier, resulting in a breast cancer diagnosis model with an accuracy exceeding 97% [20].

The purpose of this study was to develop an ML algorithm, based on the results of SF testing, to establish a PJI probability score. The objective of this model is to establish a straightforward diagnostic approach for PJI, requiring only a single SF sample annotated with appropriate biomarker results.

## Materials and methods

Study design and ethical approval

The study used prospectively collected deidentified laboratory data from SF specimens obtained to clinically diagnose PJI, as approved by the Institutional Review Board [WIRB Copernicus Group (WCG IRB)]. The study was conducted in accordance with the Transparent Reporting of Multivariable Prediction Model for Individual Prognosis or Diagnosis (TRIPOD) reporting guideline [21]. ChatGPT versions 3.5 and 4.0 (OpenAI Global, LLC, San Francisco, CA) were employed solely for copy-editing purposes in the manuscript. The tool was utilized to refine language and structure, without contributing to the generation of any new content or concepts. This study was never submitted to or evaluated by any journal other than Cureus.

Data collection and preparation

All SF specimens were obtained from patients being clinically evaluated for PJI of the hip or knee and submitted to a central clinical laboratory for comprehensive diagnostic testing (CD Laboratories, Zimmer Biomet, Towson, MD). Data from laboratory equipment were electronically transmitted to the lab information system, CGM LabDAQ (CompuGroup Medical, Austin, TX), and powered by Microsoft SQL Server. This relational database management system (RDBMS) is compliant with and adheres to industry standards. The training and test sets included laboratory data from January to September 2022. The validation set comprises data from October to December 2022. There were no disparities in the setting, eligibility criteria, predictors, and outcomes between the train/test and validation datasets.

The data were restructured from long to wide format by using Microsoft Excel Power Query, imported into a Python programming language version 3.8 (Python Software Foundation) integrated development environment (PyCharm, JetBrains, Prague, Czech Republic), and then stored as an Excel file for further analysis. Age-related data, which had been truncated to ‘>89’ for de-identification, were changed to ‘90’ for numerical analysis, and filters were applied in Python to remove samples substantially diluted with blood or with compromised integrity [22] and samples with missing biomarker data (Table 1).

**Table 1:** Inclusion and exclusion criteria This table demonstrates the number/percentage of samples that were removed from the original set due to each exclusion criterion. A single sample removed from the train/test set due to extremely high WBC was captured as missing cell count/differential *Samples missing cell count/differential were due to the following laboratory rejection rules: (1) sample not received in the required lavender top (EDTA) tube; (2) insufficient sample quantity in EDTA tube, or (3) expired EDTA tube was used. **Samples missing %PMN were due to low WBC (<1000/µL) by manual counting method, resulting in differential cell counts not being reported. ***Neutrophil elastase was used for unsupervised learning/labeling but not for the development of the decision tree model A280: optical density (absorbance) measured at 280 nanometer wavelength; PJI: periprosthetic joint infection; WBC: white blood cell concentration; %PMN: percentage of neutrophils; RBC: red blood cell concentration; N/A: not applicable; EDTA: ethylenediaminetetraacetic acid

Criteria		Train/test data	Validation data
Inclusion criteria	Patients with a hip or knee arthroplasty, n	19,764	6,453
	Comprehensive synovial fluid testing performed for PJI, n		
Exclusion criteria	Specimen substantially diluted with blood (red blood cell concentration >1,000,000/µL) or specimen integrity compromised (A280 <0.342 or A280 >1.19), n (%)	1,452 (7.3%)	470 (7.3%)
	Missing cell count/differential (WBC, %PMN, RBC)*, n (%)	825 (4.2%)	260 (4.0%)
	Missing %PMN**, n (%)	312 (1.6%)	122 (1.9%)
	Missing neutrophil elastase***, n (%)	10 (0.05%)	N/A
	Total samples utilized for model development and validation, n (%)	17,165 (86.8%)	5,601 (86.8%)

Patient age and SF biomarker test results widely regarded as diagnostic for PJI were used for model development, including absorbance at 280 nm wavelength (A280) within the range of specimen integrity [22], AD [23], C-reactive protein (SF-CRP) [24], white blood cell count (WBC) [4], polymorphonuclear cell percent (%PMN) [4], red blood cell concentration (RBC) ≤1,000,000/µL [22], human neutrophil elastase (HNE), and microbial antigen test panel (MID Panel) [13]. Feature engineering was performed to combine the five microbial antigen test results [staphylococcus panel A (SPA), staphylococcus panel B (SPB), candida panel (CP), enterococcus panel (EF), and C. acnes panel (PAC)] into a single MID Sum feature, which was included as an input feature to the model together with the individual panel members. No weighting, ranking, or clinical decision limits were applied to influence model development. SF microbiological culture (SF-Cx) was not used as an input feature to develop the model as it was used to enable classification by the 2018 ICM PJI definition.

Infection classification

A modified 2018 ICM definition of PJI was used to establish infection classification to evaluate model performance (Table 2) [4]. The 2018 ICM definition underwent modification by substituting the serum CRP with SF-CRP (threshold of 6.6 mg/L). This SF-CRP clinical decision limit has been shown to be equivalent or superior to serum CRP in the diagnosis of PJI [24,25]. The use of SF-CRP further enables contemporaneous measurement of the CRP together with the other relevant biomarkers (i.e., A280, AD, WBC, %PMN, RBC, MID Panel, HNE, and SF-Cx) from a single sample, thereby reducing the variability that can be introduced by taking multiple measurements at different times from different sample matrices.

**Table 2:** Modified 2018 ICM criteria used to establish infection classification ICM: International Consensus Meeting; WBC: white blood cell concentration; S/CO: signal to cutoff; SF-CRP: synovial fluid C-reactive protein

Condition	Score	Total score: infection classification
WBC >3000 cells/µL or alpha-defensin S/CO ≥1.00	3 points	≥6 points: infected
Neutrophils >70%	2 points	<3 points: not infected
SF-CRP ≥6.6	2 points	3–5 points: inconclusive
Positive culture result	2 points	

Using this methodology, each sample was assessed, and the results were appended as columns to the dataset: ‘Modified ICM score’ and ‘Infection Classification’. The modified ICM scores and Infection Classification labels were appended to the dataset to compare the PJI probability predictions to the diagnosis for model performance evaluation. These features were not used as inputs to the model, nor was the model trained using the modified ICM Score or the infection classification derived from it as input variables or labels.

Model development

Data Preprocessing

Using the sklearn toolkit version 1.3.0 (open-source Python package), the input features were preprocessed. Outlier handling was achieved by applying the Winsorization technique to trim at the upper 95th percentile for the following features: WBC, SPA, SPB, CP, and EF [26]. To align with the quantitative measurement range of the assay, SF-CRP values below 0.4 mg/L were trimmed to 0.4 mg/L and SF-CRP values above 60 mg/L were trimmed to 60 mg/L. A single sample with a WBC value of 44,500,000 cells/µL was removed from the dataset. Next, standard scalar was applied to age, A280, %PMN, and CP, and log transformation was applied to the remaining input features that displayed a prominent bimodal distribution: AD, SF-CRP, WBC, RBC, HNE, SPA, SPB, EF, and PAC.

Self-Directed Application of Diagnosis Labels

**Figure 1:**
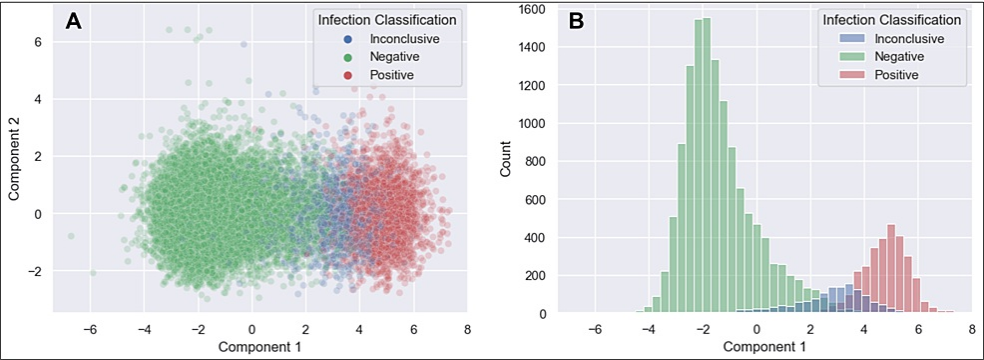
Two-dimensional principal component analysis Panel A: The image shows a two-dimensional scatter plot of the training/test dataset plotted along Component 1 and Component 2 following principal component analysis (PCA); infection classification overlayed with colored dots demonstrates this unsupervised (i.e., self-directed) approach effectively captures variability associated with infection classification. Panel B: A histogram displaying the count of samples along Component 1 further demonstrates the ability of PCA to condense infection-related variability within the dataset

**Figure 2:**
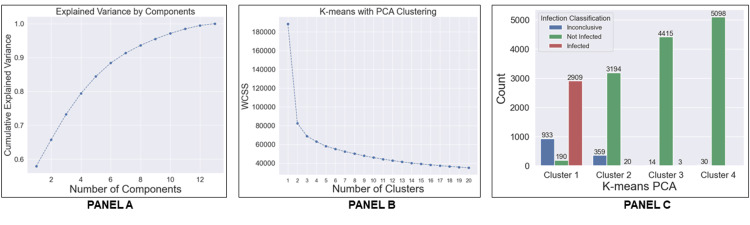
Unsupervised assignment of diagnosis labels Panel A: Unsupervised techniques were used for feature reduction via principal component analysis (PCA), followed by K-means clustering. The cumulative explained variance shows the accumulation of variance for each principal component number. Panel B: The elbow plot shows the optimum number of clusters for the entire data set after PCA transformation. Panel C: The histogram shows the number of samples classified as "infected", "not infected", and "inconclusive" clustered through the K-means algorithm WCSS: within-cluster sum of squares

The Python sklearn toolkit version 1.1.2 was used for self-directed (i.e., unsupervised) learning techniques to apply diagnosis labels to the data set with the biomarker features. First, dimensionality reduction was performed by using PCA. Overlaying the infection classification onto the samples represented by the first two principal components confirmed that unsupervised, self-directed dimensionality reduction was functioning as intended to facilitate the application of an independent label for training the predictive model (Figures 1A-1B). The explained variance ratio was used to evaluate the usefulness of the principal components and to choose how many components to carry forward in the model. Five of the components with a cumulative variance of 85% were selected to represent all features (Figure 2A). The components were then clustered using the K-means algorithm, which is a self-directed method for organizing data into groups based on similarities. The elbow method was used to find the optimal number (n=4, Figure 2B) of clusters. Each sample in the set was assigned to Clusters 1 to 4, depending on the natural segmentation of data by the clustering method. Clusters were naturally aggregated from sample data, without any other external influence. Upon the completion of the cluster assignments, it was observed that Cluster 1 consisted of samples with results typically considered to be PJI-positive by 2018 ICM criteria whereas Clusters 2-4 comprised samples with results typically considered to be PJI-negative by 2018 ICM criteria (Figure 2C). ICM "inconclusive" classification was scattered between the clusters. Hence, the self-directed cluster assignments were used to label samples in Cluster 1 as PJI-positive and samples in Clusters 2-4 as PJI-negative to train the predictive algorithm. By setting a seed, the dataset was randomized to remove temporal bias and stratified to ensure an even distribution of samples labeled as positive and negative before being split in the ratio of 80% training and 20% testing.

Supervised Approach to Predict PJI Probability

The decision tree algorithm was chosen because it offers greater interpretability for humans compared to other ML models. Additionally, it provides a probability score instead of a binary classification, enhancing its transparency and explanatory power. Our model was built with Python programming language version 3.8 (Python Software Foundation). Model training occurred in an Azure cloud-based Data Science Virtual Machine (Microsoft), which is a general-purpose standard D3_v2 machine equipped with a quad-core central processing unit, 14 GB of random-access memory, and 200 GB of storage. The decision tree was built using default parameters, which originally led to overfitting. To prevent this overfitting, post-pruning was applied after using cost complexity pruning. This pruning technique is parameterized by cost complexity parameter-alpha (ccp_alpha). The greater the value of ccp_alpha, the greater the number of nodes that are pruned. Minimal cost complexity pruning recursively finds the “weakest link” to be pruned. Five-fold cross-validation was performed during the selection of the optimal ccp_alpha value on the training set, which was established to be 0.0005.

Performance evaluation

The performance of the model created with unsupervised techniques was evaluated versus the modified 2018 ICM criteria for PJI. A receiver operating characteristic (ROC) curve was generated from the training set to identify the optimal PJI probability score that best discriminates between "infected" and "not infected" classifications. The area under the curve (AUC) was also calculated. It has been demonstrated that medical decision-making can be explained by the threshold model, whereby physicians should treat if the probability of disease exceeds a specified threshold and withhold treatment otherwise [27]. Therefore, a probability score closer to the boundary limits is advantageous for increasing diagnostic certainty. To facilitate this, the ROC data were further evaluated to identify thresholds closer to the boundaries of <1% probability and >99% probability that could be feasibly established without substantially altering the diagnostic accuracy profile. These thresholds were established as the PJI-negative decision limit and the PJI-positive decision limit to evaluate model performance.

For the evaluation of the model performance in the test and validation sets, the sensitivity, specificity, positive predictive value (PPV), and negative predictive value (NPV) were calculated. Samples with a PJI probability of greater than the PJI-positive decision limit or less than the PJI-negative decision limit were classified as positive or negative by the model, respectively. The samples between these two limits were interpreted as inconclusive by the model. To compute sensitivity and specificity, the subgroups are delineated as follows: True positive corresponds to samples classified as "infected" and predicted as PJI-positive by the model. True negative refers to samples classified as "not infected" and predicted as PJI-negative by the model. False negative designates samples classified as "infected" but predicted to be below the PJI-positive decision limit by the model. False positive pertains to samples classified as "not infected" but predicted to be above the PJI-negative decision limit by the model. Samples that had an infection classification as "inconclusive" were excluded from these analyses.

Model interpretability and discordant analysis

SHAP (Shapley Additive exPlanations) version 0.39.0 was used to explore model interpretability. SHAP is a model-agnostic method based on cooperative game theory and is used to increase the transparency and interpretability of ML models [28]. SHAP values allow local interpretability comparison by showing the feature contributions for each prediction. Waterfall plots for each prediction show how the features contribute to the predicted probability f(x) in comparison to the mean predicted probability of the model E[f(x)]. The sum of all SHAP values of the features will be equal to E[f(x)] - f(x). For this study, the absolute SHAP values show how much a single feature affects the prediction of the PJI diagnosis and which biomarkers are driving a specific instance. The SHAP values also enable global interpretability by calculating the overall feature importance across the entire dataset. The values can also be useful for understanding discrepancies between the model prediction and the infection classification, and hence it was employed for discordant analysis and to evaluate the prediction of "inconclusive" samples by the model as PJI-positive or PJI-negative.

Statistical analysis

Descriptive statistics were generated for the training/test and validation sets using scipy.stats, a sub-package of the Python package SciPy (version 1.10.1). Continuous variables were represented as median with interquartile range (IQR). Categorical variables were represented as percentages of samples in each category. Statistical comparisons were performed to evaluate for differences between the training/test and validation datasets. The t-test was used for continuous variables and the Chi-square test was used for categorical variables. GraphPad Prism 9.4.1 was used to generate a ROC curve, AUC with a 95% confidence interval (CI), and sensitivity/specificity pairs with associated Clopper-Pearson 95% CIs from the training dataset. The Youden Index was used to estimate a range of PJI probability scores associated with optimal discrimination between "infected" and "not infected" classifications. Minitab 18 was used to calculate sensitivity, specificity, PPV, and NPV using a one-sample proportion with the Exact method for 95% CI. Minitab 18 test for two proportions was used to compare the proportion of "inconclusive" results between the PJI probability score and the modified 2018 ICM Score, with the p-value calculated using the normal approximation method.

## Results

Data collection and preparation

For the training and test dataset, 19,764 hip and knee SF samples submitted to the laboratory for PJI testing from January to September 2022 were analyzed. Samples that did not meet specimen integrity requirements (RBC ≤1,000,000/µL and A280 <0.342 or A280 >1.19) were excluded (n=1,452, 7.3%). Samples without a full set of biomarker values needed for model development, and one sample with an outlier WBC result, were removed from the final train/test dataset (n=17,165, 86.8%) (Table 1). For the validation set, a total of 6,453 samples were acquired from October to December 2022. Samples that did not meet specimen integrity requirements were excluded (n=470, 7.3%). All samples with a full set of input biomarker values were retained in the dataset for validation (n=5,601, 86.8%) (Table 1).

Baseline characteristics were similar between the two datasets (Table 3). Statistically significant differences in baseline characteristics between the training/test and validation datasets were further scrutinized, revealing no clinically meaningful distinctions.

**Table 3:** Baseline characteristics *Statistically significant (p<0.05) IQR: interquartile range; A280: optical density (absorbance) measured at 280 nanometer wavelength; RBC: red blood cell concentration; SF-CRP: synovial fluid C-reactive protein; AD: alpha-defensin (signal:cutoff); %PMN: percentage of polymorphonuclear cells (i.e., % neutrophils); SPA: microbial antigen test *Staphylococcus *species panel A (signal/noise:cutoff); SPB: microbial antigen test Staphylococcus species panel B (signal/noise:cutoff); CP: microbial antigen test *Candida *species panel (signal/noise:cutoff); EF: microbial antigen test *Enterococcus *species panel (signal/noise:cutoff); PAC: microbial antigen test *Cutibacterium acnes* panel (signal/noise:cutoff); MID Sum: sum of microbial antigen test panel results; ICM: International Consensus Meeting

Variables		Training and test combined (n=17,165)	Validation (n=5,601)	P-value
Demographics				
Age, years, median (IQR)		67 (60-73)	67 (60-73)	0.790
Gender, %				<0.001*
Male		51.4%	52.0%	
Female		48.6%	47.7%	
Unknown		0.00%	0.3%	
Index joint, %				<0.001*
Right knee		48.3%	47.2%	
Left knee		43.9%	44.7%	
Right hip		4.2%	4.1%	
Left hip		3.6%	4.0%	
Biomarkers, median (IQR)				
A280		0.6 (0.5-0.8)	0.6 (0.5-0.8)	0.078
RBC, cells/µL		16,000 (6,000-50,000)	16,000 (6,000-49,000)	0.577
SF-CRP, mg/L		1.4 (0.5-4.9)	1.4 (0.5-4.7)	0.475
AD		0.1 (0.1-0.4)	0.1 (0.1-0.3)	0.024*
TNCC		691 (314-2,330)	656 (299-2,182)	0.956
%PMN		40 (26.3-66.7)	39.8 (25.9-65.1)	0.239
SF-Cx, %				<0.001*
Positive		12.8%	12.8%	
Negative		85.3%	85.2%	
Not performed		1.9%	1.9%	
Microbial antigen test panels, median (IQR)				
SPA		0.6 (0.5-0.8)	0.6 (0.5-0.9)	0.029*
SPB		0.6 (0.5-0.9)	0.6 (0.5-0.9)	0.0705
CP		0.5 (0.4-0.7)	0.5 (0.4-0.7)	<0.001*
EF		0.5 (0.5-0.6)	0.5 (0.5-0.6)	0.2502
PAC		0.1 (0.1-0.1)	0.1 (0.1-0.1)	0.312
MID Sum		2.3 (2.0-3.2)	2.3 (2.0-3.3)	0.100
Infection classification based on modified 2018 ICM, %				
PJI-positive		17.1%	16.8%	<0.001*
PJI-negative		75.1%	75.8%	
Inconclusive		7.8%	7.4%	

Infection classification with the modified 2018 ICM definition

Per the infection classification based on modified 2018 ICM Criteria, when the entire dataset was analyzed collectively, a total of 3,873 samples (17.0%) were classified as "infected", 17,143 samples (75.3%) were classified as "not infected", and 1,750 samples (7.7%) were classified as "inconclusive".

Development and performance evaluation of the predictive model

**Figure 3:**
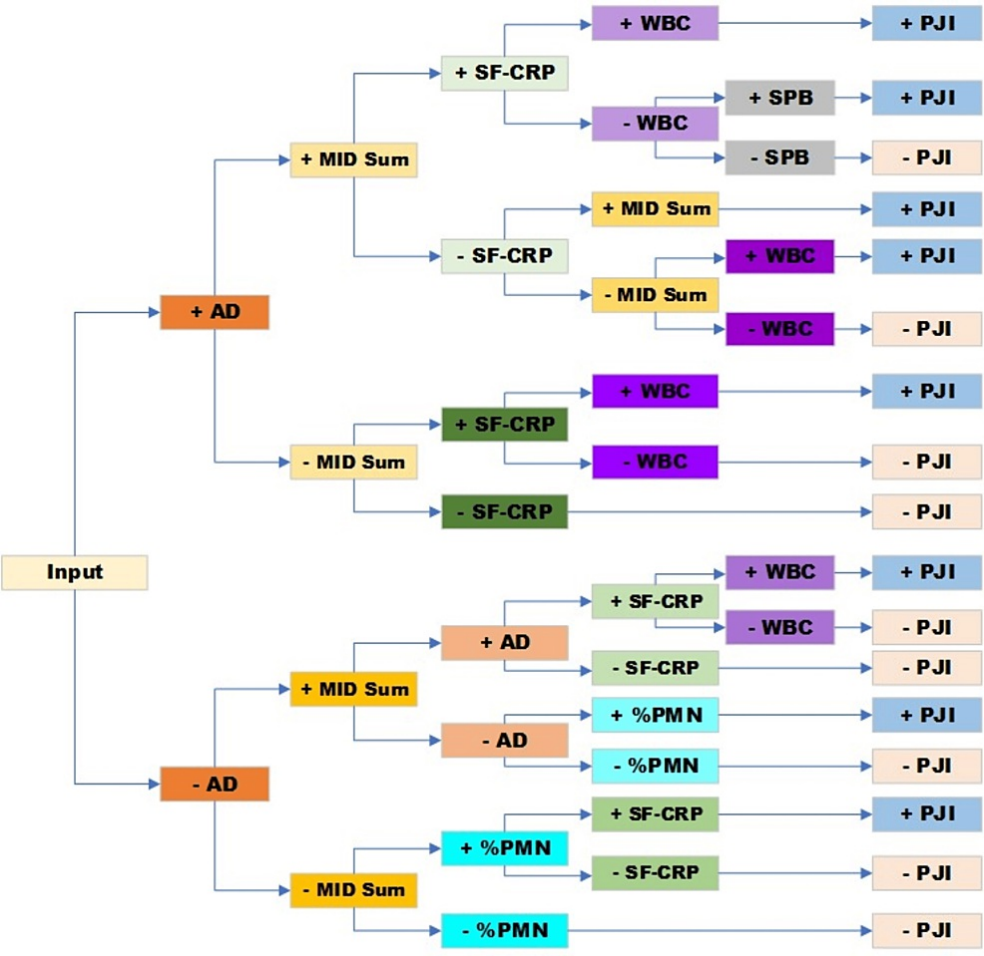
Decision tree model for classifying PJI The decision tree model establishes optimal decision thresholds for the biomarkers at each decision node, and as a result of, this some biomarkers are represented at multiple decision thresholds. Each biomarker is color-coded, with a lighter color corresponding to a lower decision threshold and a darker color corresponding to a higher decision threshold for each biomarker PJI: periprosthetic joint infection; AD: alpha-defensin; MID Sum: sum of microbial antigen test panel results; SF-CRP: synovial fluid C-reactive protein; %PMN: percentage of neutrophils; WBC: white blood cell concentration; SPB: microbial antigen test *staphylococcus* panel B

**Figure 4:**
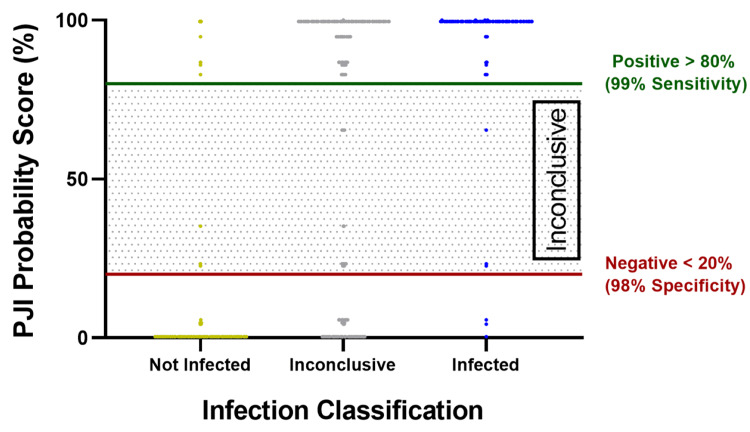
PJI probability score dot plot Receiver operating characteristic (ROC) curve sensitivity and specificity at different probability thresholds revealed that the estimated sensitivity of 99% and estimated specificity of 98% could be achieved by setting the PJI-negative decision limit to 20% and the PJI-positive decision limit to 80%. The training dataset was used for this analysis PJI: periprosthetic joint infection

The decision tree model features five layers, each comprising two nodes (Figure 3). These nodes divided the probability of PJI into two categories: PJI-positive and PJI-negative, determined by the prevailing class among the samples that fall into each respective node. ROC analysis of the training set rendered an AUC of 0.993 (95% CI: 0.991-0.995) and an optimal PJI probability score of 50% (Table 4). Further examination revealed that PJI-negative and PJI-positive decision limits could be established at 20% and 80% while maintaining the diagnostic accuracy profile (Table 4, Figure 4).

**Table 4:** Decision limits based on receiver operating characteristic curve Receiver operating characteristic (ROC) curve sensitivity and specificity at different probability thresholds revealed that the estimated sensitivity of 99% and estimated specificity of 98% could be achieved by setting the PJI-negative decision limit to 20% and the PJI-positive decision limit to 80%. The training dataset was used for this analysis CI: confidence interval; J-stat: J-statistic associated with Youden Index

Probability score	Sensitivity	95% CI	Specificity	95% CI	J-stat
>2.3%	99%	0.99 to 1.0	95%	0.95 to 0.96	0.948
>4.5%	99%	0.99 to 1.0	95%	0.95 to 0.96	0.949
>5.2%	99%	0.99 to 1.0	97%	0.97 to 0.97	0.964
>20%	99%	0.99 to 1.0	98%	0.97 to 0.98	0.968
>29%	99%	0.99 to 0.99	98%	0.98 to 0.98	0.974
>40%	99%	0.99 to 0.99	98%	0.98 to 0.99	0.976
>50%	99%	0.99 to 0.99	98%	0.98 to 0.99	0.976
>60%	99%	0.99 to 0.99	98%	0.98 to 0.99	0.976
>74%	99%	0.99 to 0.99	98%	0.98 to 0.99	0.975
>80%	99%	0.99 to 0.99	98%	0.98 to 0.99	0.975
>84%	97%	0.96 to 0.98	99%	0.98 to 0.99	0.958
>91%	95%	0.94 to 0.96	99%	0.99 to 0.99	0.943
>97%	93%	0.92 to 0.94	99%	0.99 to 0.99	0.926

**Figure 5:**
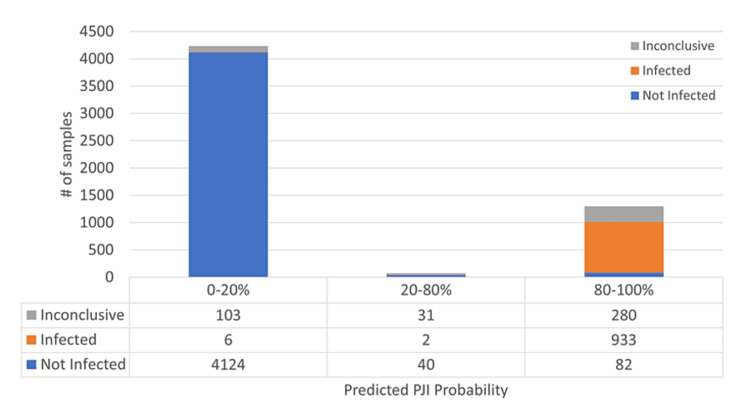
PJI probability distribution Sample distribution of predicted PJI probability score, with colors representing "not infected" (blue), "inconclusive" (gray), and "infected" (orange) classification based on modified 2018 ICM PJI: periprosthetic joint infection; ICM: International Consensus Meeting

When comparing to infection classification based on modified 2018 ICM criteria, sensitivities of 98.8% (95% CI: 97.6%-99.5%) and 99.1% (95% CI: 98.3%-99.6%), and specificities of 97.3% (95% CI: 96.7%-97.9%) and 97.1% (95% CI: 96.6%-97.6%) were achieved on the test and validation sets, respectively (Table 5). Positive and negative predictive values were 92.9% (95% CI: 90.6%-94.8%) and 99.8% (95% CI: 99.5%-99.9%) for the test set and 91.9% (95% CI: 90.1%-93.5%) and 99.9% (95% CI: 99.7%-99.9%) for the validation set (Table 5). Overall, the model predicted 1.3% of samples in the validation set as "inconclusive" (i.e., falling between 20%-80% probability score) as compared to a rate of 7.4% by ICM (p<0.001) (Figure 5), effectively reducing the Inconclusive result rate by 82.4% or more than 5X.

**Table 5:** Model performance for the test and validation sets compared to infection classification by modified 2018 ICM ICM: International Consensus Meeting; CI: confidence interval; PPV: positive predictive value; NPV: negative predictive value

	Performance characteristic	Value	95% CI
Test set (n=3,433)	Sensitivity	98.8%	97.6%-99.5%
	Specificity	97.3%	96.7%-97.9%
	PPV	92.9%	90.6%-94.8%
	NPV	99.8%	99.5%-99.9%
Validation set (n=5,601)	Sensitivity	99.1%	98.3%-99.6%
	Specificity	97.1%	96.6%-97.6%
	PPV	91.9%	90.1%-93.5%
	NPV	99.9%	99.7%-99.9%

Model interpretability and discordant analysis

Feature Importance

**Figure 6:**
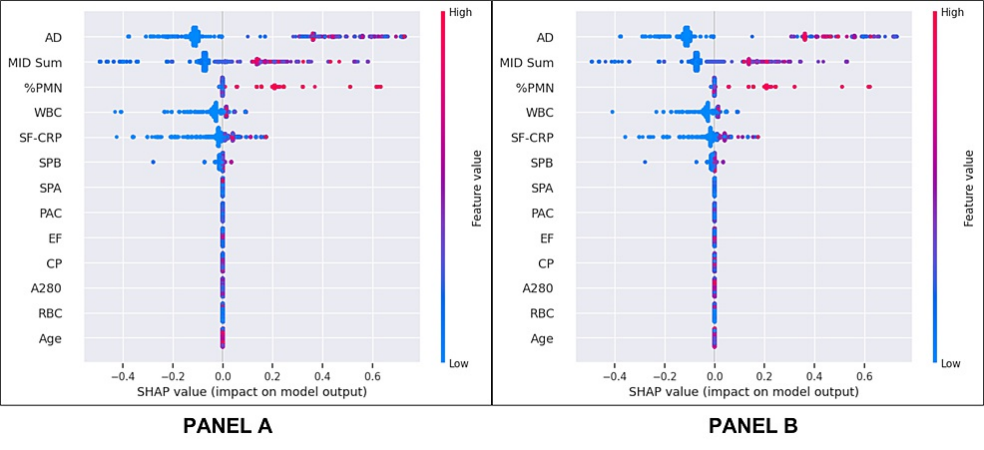
Beeswarm plot for (A) combined train/test set and (B) validation set Each variable name is shown on the left-hand side with the variable with the greatest contribution listed at the top. To the right of the variables, there are colored lines, which are individual points that correspond to the observations in the population. A higher value for the variable is represented in red, while a lower value for the variable is shown in blue. The X-axis displays the contribution of the variable to the model predictions of PJI probability for each sample, represented by dots. Hence, a value to the far right (i.e., a higher SHAP value) indicates that the variable is contributing highly to a PJI-positive prediction. As the name implies, the SHAP values are additive (or subtractive) to explain the contribution of each feature to the overall prediction SHAP: SHapley Additive exPlanations; AD: alpha-defensin; MID Sum: sum of microbial antigen test panel results; %PMN: percentage of neutrophils; WBC: white blood cell concentration; SF-CRP: synovial fluid C-reactive protein; SPB: microbial antigen test *Staphylococcus* species panel B; SPA: microbial antigen test *Staphylococcus* species panel A; PAC: microbial antigen test *Cutibacterium acnes* panel; EF: microbial antigen test *Enterococcus* species panel; CP: microbial antigen test *Candida* species panel; A280: optical density (i.e., absorbance) at 280 nanometers wavelength; RBC: red blood cell concentration

**Figure 7:**
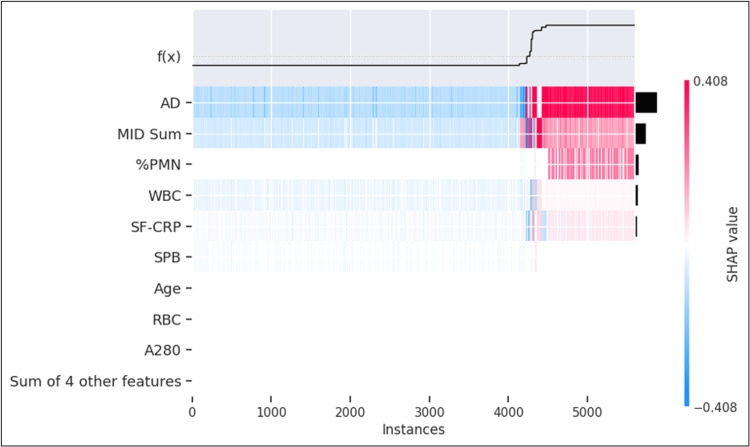
Heatmap of validation set Heatmap of all samples (i.e., instances) in the validation dataset showing SHAP values at each instance arranged in order from least to highest log odds (i.e., probability) of the sample being predicted as PJI-positive. The color scale of the heatmap is standardized to show SHAP values for the set of samples in the heatmap. The sum of individual feature SHAP values added to the mean probability of the sample set equals the PJI probability for the instance in question. Instances less than and including 4,233 have probability scores of <20% whereas instances including and greater than 4,307 have probability scores of >80% SHAP: SHapley Additive exPlanations; AD: alpha-defensin; MID Sum: sum of microbial antigen test panel results; %PMN: percentage of neutrophils; WBC: white blood cell concentration; SF-CRP: synovial fluid C-reactive protein; SPB: microbial antigen test Staphylococcus species panel B; RBC: red blood cell concentration; A280: optical density (i.e., absorbance) at 280 nanometer wavelength

In both the train/test and validation datasets, the top five features illustrated by beeswarm plots were AD, MID Sum, %PMN, WBC, and SF-CRP, and the least important features were age and the specimen integrity biomarkers (A280, RBC) (Figure 6A: train/test and Figure 6B: validation, Figure 7). In addition to being identified by SHAP as the feature with the largest effect, AD was also observed as the primary node and at several decision nodes within the decision tree (Figure 3). In the train/test set, the leading predictor of the PJI probability score was the AD result. Among samples above the 80% PJI probability threshold, a high AD result was the dominant factor that drove a positive prediction in 96% of cases, followed by MID Sum (3%), and %PMN (1%). Conversely, a low AD result was the most influential factor for 99% of samples predicted as PJI-negative (PJI probability <20%), followed by MID Sum (0.9%), and SF-CRP (0.1%).

Discordant Analysis

**Figure 8:**
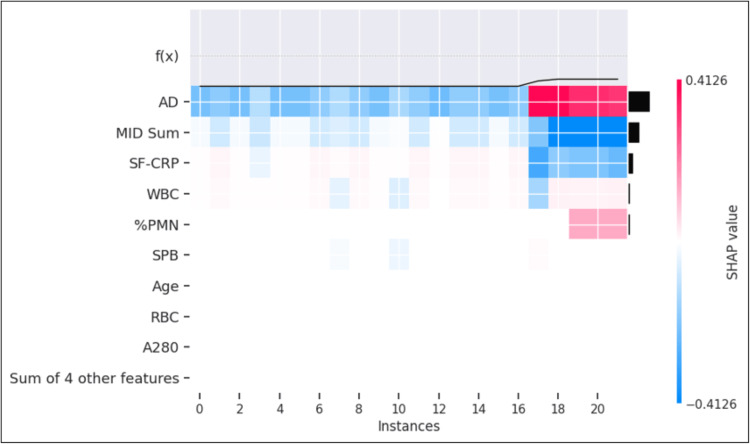
Heatmap of samples classified as "infected" and predicted as PJI-negative Discordant analysis of samples classified as "infected" and predicted as PJI-negative (maximum probability was 5.7%) by the model (n=22). The individual SHAP values in this analysis range from -0.4126 represented by dark blue to +0.4126 represented by dark red, with the color distribution centered at 0, represented by white. PJI: periprosthetic joint infection; SHAP: SHapley Additive exPlanations; AD: alpha-defensin; MID Sum: sum of microbial antigen test panel results; SF-CRP: synovial fluid C-reactive protein; WBC: white blood cell concentration; %PMN: percentage of neutrophils; SPB: microbial antigen test *Staphylococcus* species panel B; RBC: red blood cell concentration; A280: optical density (i.e., absorbance) at 280 nanometers wavelength

**Figure 9:**
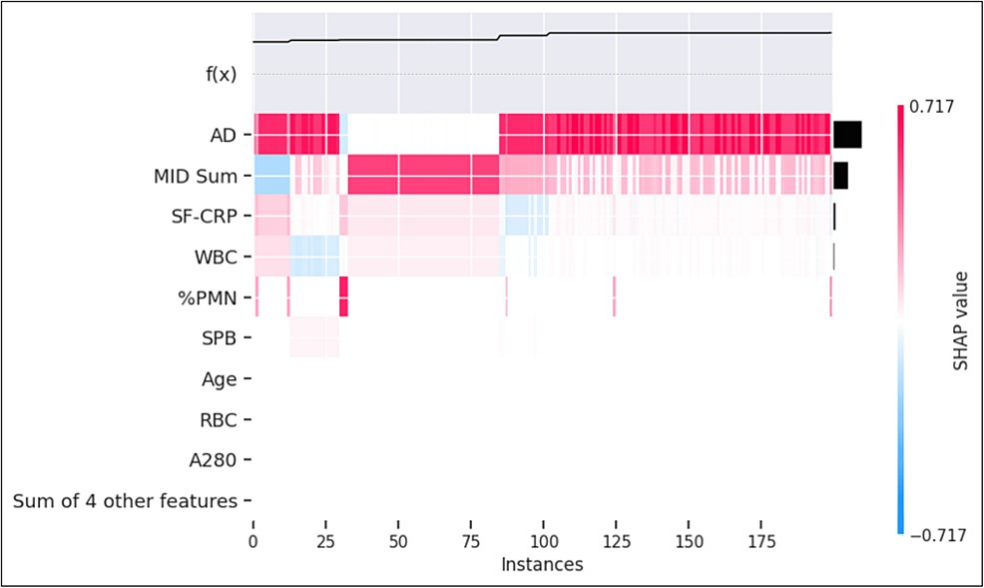
Heatmap of samples classified as "not infected" and predicted as PJI-positive Discordant analysis of samples classified as "not infected" and predicted as PJI-positive (lowest probability was 83%) by the model. The individual SHAP values in this analysis range from -0.717 represented by dark blue to +0.717 represented by dark red, with the color distribution centered at 0, represented by white PJI: periprosthetic joint infection; SHAP: SHapley Additive exPlanations; AD: alpha-defensin; MID Sum: sum of microbial antigen test panel results; SF-CRP: synovial fluid C-reactive protein; WBC: white blood cell concentration; %PMN: percentage of neutrophils; SPB: microbial antigen test Staphylococcus species panel B; RBC: red blood cell concentration; A280: optical density (i.e., absorbance) at 280 nanometers wavelength

The model prediction was 90.8% concordant with the infection classification for the validation dataset. The proportion of samples classified as "not infected" and predicted as PJI-positive was 1.5%. The proportion of samples classified as "infected" and predicted as PJI-negative was 0.1% (Figure 5). Evaluation in the larger train/test set revealed that AD was the most prominent contributor in approximately 75% of each of these cohorts (Figures 8, 9).

Evaluation of Inconclusive Sample Predictions by the Model

Within the cohort (n=414) classified as "inconclusive" by modified 2018 ICM, the model predicted 24.9% (n=103) of these as PJI-negative (PJI probability <20%), 67.6% (n=280) as PJI-positive (PJI probability >80%), and 7.5% (n=31) as "inconclusive" (PJI probability between 20%-80%) (Figure 5).

**Figure 10:**
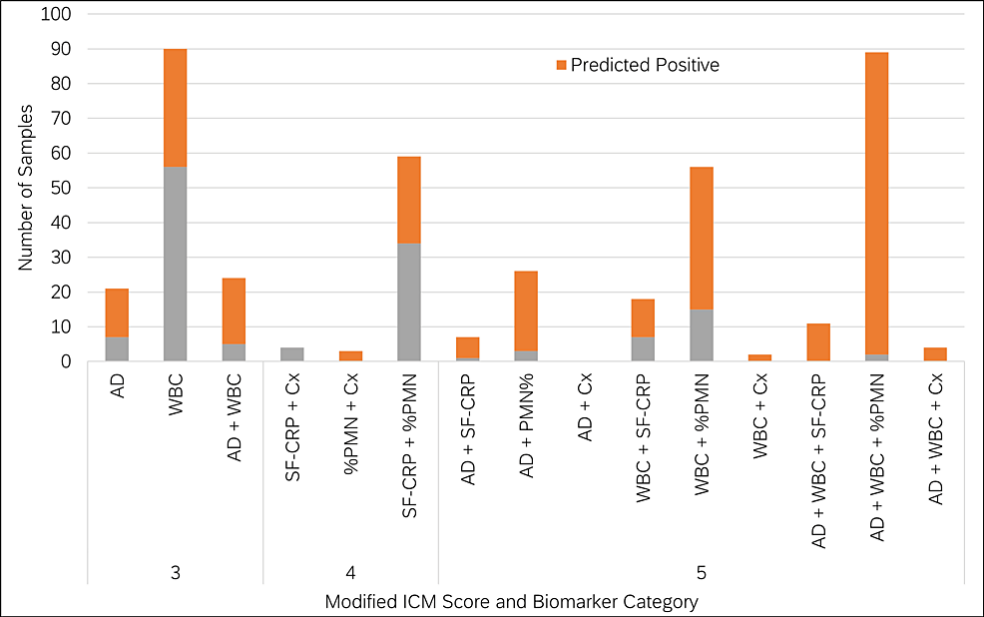
Biomarker positivity distribution for samples with "inconclusive" classification based on modified 2018 ICM This figure illustrates that samples with more positive biomarkers and samples with AD-positive are more frequently predicted as PJI-positive by the model ICM: International Consensus Meeting; PJI: periprosthetic joint infection; AD: alpha-defensin; WBC: white blood cell concentration; SF-CRP: synovial fluid C-reactive protein; Cx: microbiological culture; %PMN: percentage of neutrophils

Samples classified as "inconclusive" by modified 2018 ICM were distributed across 14 distinct biomarker positivity categories using the modified 2018 ICM thresholds (Table 2). The largest category consisted of a cohort exhibiting solely a positive WBC biomarker, with a modified 2018 ICM score of 3 points. Among these 90 samples, the model predicted 38% as PJI-positive. The second largest category comprised a cohort with positive results for AD, WBC, and %PMN, possessing a modified 2018 ICM score of 5 points. Out of these 89 samples, the model predicted 98% as PJI-positive. Additionally, the model predicted positive outcomes for over 98% of samples with three positive biomarkers (Figure 10). It was also observed that microbiological culture (Cx) was positive in only 3.1% (13 of 414) of this cohort. The model predicted 69% (9 of 13) of these as PJI-positive. Notably, all Cx-positive samples that the model did not predict as positive belonged to the same biomarker positivity category (SF-CRP and Cx).

**Figure 11:**
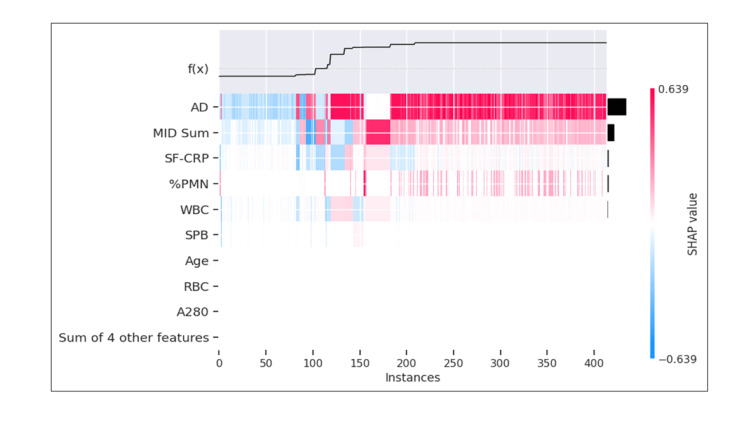
Model prediction of samples with "inconclusive" classification based on modified 2018 ICM The heatmap shows SHAP values at each instance, arranged in order from least to highest probability of samples being predicted as PJI-positive. The color scale is standardized to show SHAP values for the set of samples in the heatmap. The sum of individual feature SHAP values added to the mean probability of the sample set equals the PJI probability for the instance in question. The individual SHAP values in this analysis range from -0.639 represented by dark blue to +0.639 represented by dark red, with the color distribution centered at 0, represented by white ICM: International Consensus Meeting; PJI: periprosthetic joint infection; SHAP: SHapley Additive exPlanations; AD: alpha-defensin; MID Sum: sum of microbial antigen test panel results; SF-CRP: synovial fluid C-reactive protein; %PMN: percentage of neutrophils; WBC: white blood cell concentration; SPB: microbial antigen test *Staphylococcus* species panel B; RBC: red blood cell concentration; A280: optical density (i.e., absorbance) at 280 nanometer wavelength

Feature importance pertaining to model prediction of the samples classified as "inconclusive" by modified 2018 ICM was explored (Figure 11). The heatmap shows that AD was the most important feature for 87% of these samples. AD was driving both low-negative and high-positive predictions.

## Discussion

This study leverages an extensive SF dataset to develop an ML model for aiding the diagnosis of PJI. Central to this research is the unsupervised clustering of samples into distinct, naturally occurring categories. These clusters, which emerged organically within the dataset, form the foundation for subsequent analyses. A decision tree model was then developed, utilizing these clusters as labels for training new sample classifications, distinct from the original clustering. The model was initially trained on a designated training set and further evaluated against a test set derived from the same initial population. To validate the model, a separate validation cohort was employed, enabling a critical assessment of its performance against the modified 2018 ICM definition of PJI, where it demonstrated notable diagnostic accuracy. Additionally, the application of the SHAP method underscored the importance of key biomarkers, such as AD, MID Sum, %PMN, and SF-CRP, in the model’s decision-making. This model, by processing and integrating diverse diagnostic data, offers a potentially more efficient and accurate alternative to the manual compilation, interpretation, and scoring of multiple tests which is currently performed by individual clinicians. We propose that the integration of such a model into the laboratory reporting of PJI testing would reduce human error in diagnosing infection, translating into a higher rate of appropriate patient treatment plans and improved patient outcomes.

The unsupervised clustering of SF samples into naturally occurring categories is a pivotal aspect of this study. This approach, devoid of preconceptions about PJI, analyzes the inherent structures within the dataset to categorize samples based on feature set similarities. The dataset, encompassing 17,165 samples with over 12 potential feature inputs, presented a significant challenge in developing an accurate predictive model due to its high-dimensionality metadata [17]. To address this, we utilized principal component analysis and unsupervised K-means clustering to autonomously organize data and reduce redundancy in features. While unsupervised ML has previously been used to stratify patients based on PJI risk [29], this study represents a novel application of these methods to a large SF dataset, creating clinically meaningful diagnostic subgroups. We identified four distinct clusters in our dataset. Assessing these clusters against the 2018 ICM scoring revealed three clusters containing ICM-negative samples and one cluster with ICM-positive samples. Notably, samples classified as "inconclusive" by the modified 2018 ICM definition were distributed across several clusters.

Building on these unsupervised clusters, an ML algorithm was developed to classify samples. A decision tree model, selected for its clarity and interpretability, was trained to align with the unsupervised clustering. The diagnostic model's evaluation against a modified 2018 ICM definition of PJI yielded an AUC of 0.993, with a sensitivity of 99.1% and a specificity of 97.1% in the unlabeled, independent validation dataset. The alignment between the ML classification and the modified 2018 ICM definition underscores the clinical relevance of the unsupervised clustering outcomes. Interestingly, while the modified 2018 ICM definition applied to this study's validation samples resulted in a rate of "inconclusive" diagnoses of 7.4%, the ML algorithm reduced this rate to 1.3%.

The final ML decision tree was analyzed using the SHAP method. This approach ensured an unbiased evaluation process, free from any preferential treatment of specific biomarkers. The analysis revealed that the AD test result emerged as the most dominant feature, aiding in the decision-making process for over 95% of samples. This finding aligns with existing literature including a meta-analysis, where AD has been recognized as one of the most accurate biomarkers for PJI [30]. Equally noteworthy was the identification of the MID Sum panel as the second most influential feature in the model. This observation suggests that the combination of a key inflammatory marker with a dominant organism detection feature was instrumental in sample stratification. The MID panel, encompassing various antigen tests for common PJI pathogens, has been previously noted for its high positive and negative predictive value in PJI diagnosis [13]. Also, the neutrophil percentage, white blood cell concentration, and SF-CRP results were in the top five dominant biomarkers, all known to be important determinants of the diagnosis of PJI.

In the evolving landscape of PJI diagnostics, the integration of ML models presents an opportunity to enhance the accuracy and efficiency of clinical decision-making. The complexity inherent in the current standard of care, particularly with the 2018 ICM definition, necessitates meticulous attention to detail in test ordering, data conversion, and score computation. This process is susceptible to human error [9,10] and the misapplication of scoring thresholds [12], potentially affecting diagnostic accuracy. Even researchers with subject matter expertise have misapplied the scoring thresholds, leading to inaccuracies in categorizing infections. In contrast, the proposed ML model automates these steps, effectively reducing the likelihood of error. Decreasing errors in the diagnosis of PJI could have a substantial impact on patient care. Given the severity of PJI, its diagnosis triggers a completely different treatment plan for the patient. When the diagnosis is negative, a simple revision or observation of the patient frequently suffices as the preferred treatment plan. On the other hand, a positive diagnosis of PJI generally requires urgent surgery, which often includes multiple stages, requires high-dose intravenous antibiotics, and has inferior results even when eradicating infection [31]. The diagnosis of PJI is therefore critical for both the treatment plan chosen by the clinician and the outcome experienced by the patient. Furthermore, given the high cost of revision joint surgery for PJI, even small to moderate reductions in human error would be expected to result in cost savings [32].

Another critical aspect where the ML model holds an advantage is in reducing diagnostic ambiguity. One of the challenges with the current standard of care is the relatively high rate of physician uncertainty and inconclusive diagnoses, which can lead to clinical delays in providing appropriate treatment. The ML model addresses this issue by significantly lowering the rate of inconclusive results, thus providing clearer and more decisive diagnostic outcomes. This reduction in ambiguity is pivotal in clinical settings, where timely and confident decision-making is essential. The existing literature suggests that delays in the care of PJI may lead to poor patient outcomes, even when the correct treatment is eventually rendered [33]. Therefore, an ML model increasing diagnostic certainty and streamlining the diagnostic process would be expected to lead to faster physician decision-making and hence an improvement in patient outcomes.

Finally, the dynamic nature of medical guidelines, especially in the context of PJI, presents a significant challenge in clinical practice. New definitions and guidelines are frequently introduced, but their adoption into clinical practice often encounters a lag, primarily due to the time it takes for physicians to familiarize themselves with these changes [34]. The ML model tackles this challenge by facilitating real-time updates of newly validated enhancements and data, emphasizing a dynamic approach to address evolving needs. This capability significantly shortens the adoption curve, ensuring that physicians are always working with the most current standards without the need for extensive retraining or knowledge updates. Such a feature is particularly advantageous in the rapidly evolving field of medical diagnostics, where staying abreast of the latest developments is crucial for optimal patient care [35].

Limitations

The study has a few limitations that warrant discussion. First, it entails a report of model performance in a base case example. Samples with missing data for any of the biomarkers used as features in the model development were removed. Similarly, samples failing to meet specimen integrity requirements of the laboratory were omitted, and only samples from hip or knee arthroplasty were included in order to align with the applicability of the 2018 ICM definition. Consequently, the model performance for samples with a missing feature, poor integrity, or alternative joint location (e.g., shoulder, wrist) is unknown. This limitation is not unique to this model and does not undermine the performance results, particularly since these omissions accounted for less than 15% of the specimens tested at the laboratory. Certainly, attempting to make a diagnosis with missing information or interpreting potentially compromised results from in vitro diagnostic testing performed with a poor-quality sample are circumstances that should be avoided; nonetheless, these events do arise in the real-world application of medicine. Future work should evaluate these edge cases to further our understanding of how model performance may be impacted by missing inputs, specimen dilution or contamination, and anatomical location.

Secondly, the model has not been evaluated on data from samples tested by another laboratory; hence, further validation work would be necessary to put this model into use elsewhere. Third, the results of a decision-tree algorithm, even though easy to understand and interpret, may be prone to overfitting and instability and may show differences in performance in case of a data drift [36]. Notably, there are risk and benefit factors that support model selection, as all models have strengths and weaknesses. In this case, the limitations associated with a decision-tree algorithm are mitigated by access to a large amount of very recent data [37]. Finally, only preoperative SF biomarker data was available to classify samples using the 2018 ICM criteria. While the authors of the 2018 ICM criteria [4] indicated that “not all tests are needed to use this proposed definition, and a preoperative diagnosis can be made without the need for intraoperative findings,” the use of preoperative SF data for diagnosis could be viewed as both a limitation and a strength of this study. The focus on the preoperative assessment in this study highlights the potential for this PJI probability score to enhance preoperative diagnosis of infection, which is crucial when surgeons need to make definitive treatment decisions.

## Conclusions

Although defined methods and tools for the diagnosis of PJI using multiple biomarker criteria are currently available, they are not consistently applied or broadly adopted. There is a need for algorithmic interpretation of these biomarkers to enable consistent application of the results to drive treatment decisions. The new model developed to apply to clinical parameters measured from a patient’s SF sample to render a preoperative probability score for PJI has shown high accuracy. Taken together with other clinical signs, this model has the potential to improve the diagnostic process by reducing the rate of Inconclusive classification, thereby enabling more appropriate and expedited downstream treatment decisions.

## References

[1] Mu W, Ji B, Cao L (2023). Single-stage revision for chronic periprosthetic joint infection after knee and hip arthroplasties: indications and treatments. Arthroplasty.

[2] Parvizi J, Zmistowski B, Berbari EF (2011). New definition for periprosthetic joint infection: from the Workgroup of the Musculoskeletal Infection Society. Clin Orthop Relat Res.

[3] Parvizi J, Gehrke T (2014). Definition of periprosthetic joint infection. J Arthroplasty.

[4] Shohat N, Bauer T, Buttaro M (2019). Hip and knee section, what is the definition of a periprosthetic joint infection (PJI) of the knee and the hip? Can the same criteria be used for both joints?: Proceedings of International Consensus on Orthopedic Infections. J Arthroplasty.

[5] McNally M, Sousa R, Wouthuyzen-Bakker M (2021). The EBJIS definition of periprosthetic joint infection. Bone Joint J.

[6] Osmon DR, Berbari EF, Berendt AR (2013). Diagnosis and management of prosthetic joint infection: clinical practice guidelines by the Infectious Diseases Society of America. Clin Infect Dis.

[7] Lugtenberg M, Zegers-van Schaick JM, Westert GP, Burgers JS (2009). Why don't physicians adhere to guideline recommendations in practice? An analysis of barriers among Dutch general practitioners. Implement Sci.

[8] Barth JH, Misra S, Aakre KM, Langlois MR, Watine J, Twomey PJ, Oosterhuis WP (2016). Why are clinical practice guidelines not followed?. Clin Chem Lab Med.

[9] Forte SA, D'Alonzo JA, Wells Z, Levine B, Sizer S, Deirmengian C (2022). Laboratory-reported normal value ranges should not be used to diagnose periprosthetic joint infection. Cureus.

[10] Deirmengian C, McLaren A, Higuera C, Levine BR (2022). Physician use of multiple criteria to diagnose periprosthetic joint infection may be less accurate than the use of an individual test. Cureus.

[11] Kuo FC, Hu WH, Hu YJ (2022). Periprosthetic joint infection prediction via machine learning: comprehensible personalized decision support for diagnosis. J Arthroplasty.

[12] Sigmund IK, Luger M, Windhager R, McNally MA (2022). Diagnosing periprosthetic joint infections: a comparison of infection definitions: EBJIS 2021, ICM 2018, and IDSA 2013. Bone Joint Res.

[13] Toler KO, Paranjape PR, McLaren A, Levine B, Ong A, Deirmengian C (2023). Nationwide results of microorganism antigen testing as a component of preoperative synovial fluid analysis. J Bone Joint Surg Am.

[14] Jayatilake SM, Ganegoda GU (2021). Involvement of machine learning tools in healthcare decision making. J Healthc Eng.

[15] An Q, Rahman S, Zhou J, Kang JJ (2023). A comprehensive review on machine learning in healthcare industry: classification, restrictions, opportunities and challenges. Sensors (Basel).

[16] Maxim LD, Niebo R, Utell MJ (2014). Screening tests: a review with examples. Inhal Toxicol.

[17] Omuya EO, Okeyo GO, Kimwele MW (2021). Feature selection for classification using principal component analysis and information gain. Expert Syst Appl.

[18] Ghomroudi PA, Scaltritti M, Grecucci A (2023). Decoding reappraisal and suppression from neural circuits: a combined supervised and unsupervised machine learning approach. Cogn Affect Behav Neurosci.

[19] Ahsan MM, Luna SA, Siddique Z (2022). Machine-learning-based disease diagnosis: a comprehensive review. Healthcare (Basel).

[20] Zheng B, Yoon SW, Lam SS (2014). Breast cancer diagnosis based on feature extraction using a hybrid of K-means and support vector machine algorithms. Expert Syst Appl.

[21] Collins GS, Reitsma JB, Altman DG, Moons KG (2015). Transparent reporting of a multivariable prediction model for individual prognosis or diagnosis (TRIPOD): the TRIPOD Statement. BMC Med.

[22] Deirmengian C, Feeley S, Kazarian GS, Kardos K (2020). Synovial fluid aspirates diluted with saline or blood reduce the sensitivity of traditional and contemporary synovial fluid biomarkers. Clin Orthop Relat Res.

[23] Frangiamore SJ, Gajewski ND, Saleh A, Farias-Kovac M, Barsoum WK, Higuera CA (2016). α-Defensin accuracy to diagnose periprosthetic joint infection-best available test?. J Arthroplasty.

[24] Tetreault MW, Wetters NG, Moric M, Gross CE, Della Valle CJ (2014). Is synovial C-reactive protein a useful marker for periprosthetic joint infection?. Clin Orthop Relat Res.

[25] Parvizi J, McKenzie JC, Cashman JP (2012). Diagnosis of periprosthetic joint infection using synovial C-reactive protein. J Arthroplasty.

[26] Hamadani A, Ganai NA (2023). Artificial intelligence algorithm comparison and ranking for weight prediction in sheep. Sci Rep.

[27] Djulbegovic B, Elqayam S, Reljic T (2014). How do physicians decide to treat: an empirical evaluation of the threshold model. BMC Med Inform Decis Mak.

[28] Prendin F, Pavan J, Cappon G, Del Favero S, Sparacino G, Facchinetti A (2023). The importance of interpreting machine learning models for blood glucose prediction in diabetes: an analysis using SHAP. Sci Rep.

[29] Lu Y, Salmons HI, Mickley JP, Bedard NA, Taunton MJ, Wyles CC (2023). Defining clinically meaningful subgroups for risk stratification in patients undergoing revision total hip arthroplasty: a combined unsupervised and supervised machine learning approach. J Arthroplasty.

[30] Bonanzinga T, Ferrari MC, Tanzi G, Vandenbulcke F, Zahar A, Marcacci M (2019). The role of alpha defensin in prosthetic joint infection (PJI) diagnosis: a literature review. EFORT Open Rev.

[31] Li C, Renz N, Trampuz A (2018). Management of periprosthetic joint infection. Hip Pelvis.

[32] Premkumar A, Morse K, Levack AE, Bostrom MP, Carli AV (2018). Periprosthetic joint infection in patients with inflammatory joint disease: Prevention and diagnosis. Curr Rheumatol Rep.

[33] Bedair HS, Katakam A, Bedeir YH, Yeroushalmi D, Schwarzkopf R (2020). A decision analysis of treatment strategies for acute periprosthetic joint infection: early irrigation and debridement versus delayed treatment based on organism. J Orthop.

[34] Beauchemin M, Cohn E, Shelton RC (2019). Implementation of clinical practice guidelines in the health care setting: a concept analysis. ANS Adv Nurs Sci.

[35] Adler-Milstein J, Aggarwal N, Ahmed M (2022). Meeting the moment: addressing barriers and facilitating clinical adoption of artificial intelligence in medical diagnosis. NAM Perspect.

[36] Sahiner B, Chen W, Samala RK, Petrick N (2023). Data drift in medical machine learning: implications and potential remedies. Br J Radiol.

[37] Chen JH, Alagappan M, Goldstein MK, Asch SM, Altman RB (2017). Decaying relevance of clinical data towards future decisions in data-driven inpatient clinical order sets. Int J Med Inform.

